# Clinical disease in British sheep infected with an emerging strain of bluetongue virus serotype 3

**DOI:** 10.1002/vetr.4910

**Published:** 2024-12-16

**Authors:** Kerry Newbrook, Emmanuel Obishakin, Laura A. Jones, Ryan Waters, Martin Ashby, Carrie Batten, Christopher Sanders

**Affiliations:** ^1^ The Pirbright Institute Woking UK

**Keywords:** bluetongue virus serotype 3, bluetongue, BTV‐3, sheep

## Abstract

**Background:**

Bluetongue virus serotype 3 (BTV‐3) was detected for the first time in cattle and sheep in southern England in 2023, the first UK BTV incursion for more than 15 years. Clinical signs were not observed, yet severe clinical disease and mortality were reported during recent BTV‐3 outbreaks in northern Europe.

**Methods:**

To investigate the clinical disease and infection kinetics associated with this UK BTV‐3 strain, five British sheep were infected with a UK BTV‐3 isolate using *Culicoides* biting midges. Clinical signs, pathology, infection dynamics, immune responses and *Culicoides* infection rates were assessed.

**Results:**

All sheep were infected with BTV‐3 and developed mild to moderate clinical bluetongue disease, characterised by fever, haemorrhagic diarrhoea, lameness, depression and widespread petechial haemorrhage. Three sheep reached clinical humane endpoints and were euthanased. Clinical signs/severity, infection kinetics and immune responses were highly variable. Infectious BTV‐3 was isolated from sheep blood up to 28 days postinfection.

**Limitations:**

The impact of BTV‐3 infection on British cattle and infection rate in UK *Culicoides* require investigation to fully determine the risk of this strain to UK livestock.

**Conclusions:**

This study confirms the potential impact of a BTV‐3 incursion/outbreak on the UK sheep population, highlighting the need for an effective vaccine.

## INTRODUCTION

Bluetongue (BT) is an economically important, infectious haemorrhagic disease of domestic and wild ruminants, including sheep, cattle, goats, deer and camelids. It is caused by infection with bluetongue virus (BTV), an *Orbivirus* (Sedoreoviridae) transmitted predominantly by *Culicoides* biting midges (Diptera: Ceratopogonidae).

BT is characterised by facial oedema, fever, conjunctivitis, breathing difficulties, reddening/petechial haemorrhage of mucosal membranes, nasal discharge, depression and lameness.^1^ BT is often most clinically severe in sheep, particularly European fine wool and mutton breeds, and can devastate livestock production and restrict movement and trade. Given its global economic burden, BT is notifiable to the World Organisation for Animal Health (BTV serotypes 1‒24 only). Suspected BT disease in the UK, by law, must be reported immediately to the APHA.

BTV (serotype 8; BTV‐8) was first detected in northern Europe in 2006, emerging in the UK for the first time in 2007. Following an effective BTV‐8 vaccination campaign, the UK was declared free from BTV in 2011.^2^, ^3^ However, over the last decade, there have been incursions of several BTV strains across Europe and a BTV‐8 strain is endemic in France.^4^, ^5^, ^6^, ^7^

A novel BTV serotype 3 strain (BTV‐3 NET2023) emerged in northern Europe for the first time in September 2023 from an unknown origin.^8^ It shared high genetic sequence identity with past BTV‐3 outbreak strains in Italy and Tunisia, although only across some BTV genome segments.^8^ It was first detected, and spread rapidly, in the Netherlands, followed by Belgium and Germany.^4^, ^8^, ^9^ There have been over 7000 confirmed cases in the Netherlands alone, with clinical BT disease observed in sheep, cattle and goats.^8^ The Netherlands reported severe clinical disease in sheep and production losses (especially reduced milk yield) in cattle, with 3.9% mortality in sheep and 0.2% mortality in cattle reported across the national herd.^4^ In Dutch flocks with confirmed BTV‐3 infection, mortality was up to 12.8 (lambs) and 15.1 (adult sheep) times greater than for other flocks within BTV‐3 areas.^10^ Clinical signs of fever, lethargy, hypersalivation, ulcerations/erosions of nasal/oral mucosa, facial oedema, coronary band lesions, lameness and mortality were observed in BTV‐3‐infected sheep, with some cattle also presenting with conjunctivitis, nasal discharge and oedema, lip/nostril crusting, superficial teat necrosis and coronitis.^8^

On 10 November 2023, during annual active surveillance on the southern coast of England, BTV was detected in a cow from Kent.^4^ This was the first detected UK incursion of BTV since 2007. Next‐generation sequencing confirmed that the BTV strain shared 99.9% or more nucleotide sequence identity with the emergent Netherlands BTV‐3 strain. Following active surveillance, and reactive surveillance within temporary control zones around affected farms, 119 cattle and seven sheep were found to have been infected with BTV‐3 across four counties (73 locations) in the south of England (Kent, Norfolk, Suffolk and Surrey).^4^ In all cases, no clinical signs of BT were observed at the time of sampling (or reported prior). The emergence of BTV‐3 in the UK is thought to have resulted from windborne incursion of infected *Culicoides* from northern Europe. Given the severity of clinical BT observed in Europe, UK livestock (particularly on southeastern borders) are considered at high risk should there be another incursion or re‐emergence of this BTV‐3 strain. Understanding the likely clinical impact of this emerging BTV‐3 strain on UK sheep flocks, its infection kinetics and host immune responses will aid in the development of efficacious vaccines and improved diagnostics. Defining specific clinical signs will ensure robust frontline reporting of suspected BT cases, a vital surveillance activity of the UK veterinary and farming sectors.

In this study, we infected five British sheep with the UK BTV‐3 isolate to assess the resulting clinical signs, pathology, infection kinetics, antibody responses and onwards transmission potential to a *Culicoides* vector. We also aimed to highlight possible challenges for livestock owners and veterinarians in recognising clinical signs of infection.

## MATERIALS AND METHODS

### Virus

BTV‐3 UKG2023/04 was originally isolated from the blood of a 6‐year‐old female Simmental beef cow in Norfolk, UK, in December 2023 following two serial passages in KC cells. The tissue culture supernatant (TCS) was serially passaged twice more in KC cells (KC4) to obtain a suitable infectious viral titre, as determined by titration.^11^ Next‐generation sequencing, as performed previously,^12^ confirmed 99.9% or more nucleotide sequence identity across the BTV genome between the blood isolate and KC4 TCS.

### Insects

Newly emerged adult *Culicoides sonorensis* midges (PIRB‐S‐3 strain^13^) were obtained from the AA colony line^14^ at The Pirbright Institute.^15^ Insects were housed in netted card pots (Watkins and Doncaster) maintained at 25°C (70%‒90% relative humidity) with ad libitum access to 10% (w/v) sucrose solution on cotton wool.

### Cells

KC cells derived from 2‐day‐old *C. sonorensis* embryos^16^ were maintained as previously described.^17^

### In vivo study

Six adult (>6 years) female North Country mule sheep (five Swaledale × Leicester [sheep one, two, four, five, six], one Suffolk × North Country mule [sheep three]) were used in the study, following 7‐day acclimatisation, which was performed at The Pirbright Institute's high‐containment animal facility. All sheep tested negative for BTV RNA (quantitative real‐time RT‐PCR) and serum BTV viral protein 7 (VP7) antibodies (ELISA) before the study. Sheep were group‐housed, free roaming and fed ad libitum hay and water, supplemented with grain pellets.

**FIGURE 1:**
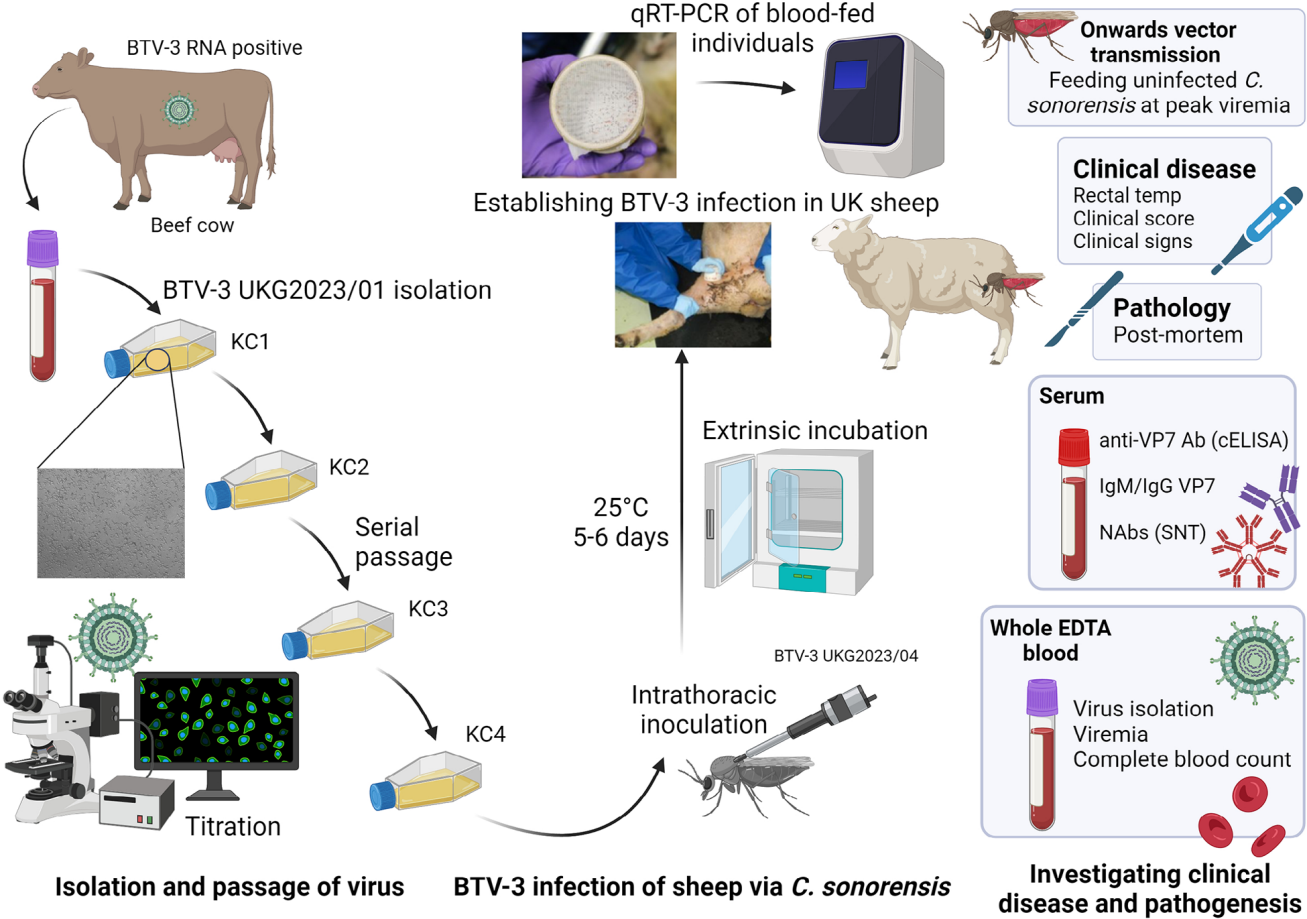
Investigating bluetongue virus serotype 3 (BTV‐3) strain UKG2023 infection in British sheep using a natural host‒virus‒vector transmission model. BTV‐3 was isolated from an infected cow during the 2023 UK incursion and serially passaged through KC cells (derived from *Culicoides sonorensis*) to achieve a suitable infectious viral titre (determined by titration). *C. sonorensis* midges were intrathoracically inoculated with BTV‐3 UKG2023 and then incubated for 5‒6 days (25°C; extrinsic incubation period) to enable viral dissemination to the salivary gland, after which they were allowed to feed on sheep to establish BTV‐3 infection. Engorged and partially engorged *Culicoides* midges were collected to determine the number of transmissibly infected midges that had blood fed on each sheep using RNA extraction and BTV quantitative real‐time RT‐PCR. Clinical signs, gross pathological changes, viraemia kinetics, host immune responses to infection and onwards transmission potential to a susceptible *Culicoides* vector were assessed. Created with Biorender.com

BTV‐3 infection was established in five sheep, the number of which was averaged from previous BTV infection studies,^1^, ^18^, ^19^ using a natural in vivo host‒virus‒vector transmission model (Figure 1) using *C. sonorensis* midges.^20^ One sheep was assigned as an uninfected control (based on skin injury on arrival) to monitor for contact transmission. Due to their low competence for this BTV‐3 strain (Supporting Information S1), *C. sonorensis* were intrathoracically inoculated with 0.2 µL BTV‐3 UKG2023/04 [KC4] suspension (10^7^ TCID_50_ per millilitre), as previously described.^15^ The inoculated *C. sonorensis* were incubated in card pots (65‒70 individuals per pot) for 5 or 6 days at 25°C, and then two pots were held on the inner thigh skin of each sheep for 10 minutes to allow the insects to blood feed. The number of infectious bites each sheep received and the infection status of blood‐fed *C. sonorensis* were determined as previously described.^11^, ^15^ Uninfected *C. sonorensis* were blood fed on each sheep at peak viraemia to investigate BTV infection rate as an indicator of onwards transmission, as previously described.^11^, ^15^

Whole EDTA and clotted blood samples were collected from the jugular vein of each animal on the day before infection and at 2, 3 (EDTA only), 4, 5, 6, 7, 8, 9, 10, 12, 14, 16, 18, 20, 22, 24, 26 and 28 days postinfection (dpi). The study was conducted under a Home Office project licence with moderate severity limits, using humane endpoints refined from previous studies of different BTV strains at The Pirbright Institute.^11^, ^15^, ^21^ Sheep were euthanased at the end of the study (28 dpi), or upon reaching their clinical humane endpoint, by intravenous administration of an anaesthetic overdose (140 mg/kg pentobarbital sodium) followed by confirmation of permanent cessation of circulation. A postmortem examination was carried out on each animal, with gross abnormalities recorded. Clinical signs were recorded daily, rectal temperatures were recoded twice daily and total clinical scores were determined for each sheep, as previously described.^11^ Sampling/treatment confounders were not specifically controlled in this study, and blinding was considered impracticable given the low availability of experienced animal technicians. Serum was collected from centrifuged clotted blood (2000 *g*, 10 minutes, 4°C).

### RNA extraction

Total RNA was extracted from 100 µL of EDTA blood, tissue homogenate or midge homogenate using a MagMax CORE nucleic acid purification kit and KingFisher Flex automated extraction robot (Thermo Fisher Scientific) according to the manufacturer's instructions. Tissues (20% [w/v] in PBS) were homogenised for 3 minutes, as previously described.^22^

### Quantitative real‐time RT‐PCR

BTV segment 10 RNA was amplified using EXPRESS One‐Step Superscript quantitative real‐time RT‐PCR kit (Thermo Fisher Scientific) as previously described,^23^ with modification. Five microlitres of RNA was denatured (95°C, 5 minutes), and 15 µL of reaction mix (comprising 1× reaction mix, 400 nM forward and reverse primers, 200 nM probe, 0.4 µL ROX passive reference dye and 2 µL EXPRESS SuperScript Mix) was added. An Applied Biosystems QuantStudio 5 Real‐Time PCR System (Thermo Fisher Scientific) was used under the following conditions: 50°C for 15 minutes; 95°C for 20 seconds; and 45 cycles of 95°C for 3 seconds, 56°C for 30 seconds and 72°C for 30 seconds. A 10‐fold serial dilution of BTV‐1 segment 10 RNA transcript^24^ was run alongside RNA from each extraction to quantify BTV genome copies per millilitre of blood.

### Virus isolation

EDTA blood was washed three times (1× PBS) and then sonicated at 60% amplitude for 15 seconds (pulse; 5 seconds on/off) with a Qsonica cup horn 431C2 and Fisherbrand Model 505 sonic dismembranator (Fisher Scientific UK). Virus isolation was attempted through the passage of 200 µL of sonicated blood (diluted in 1.8 mL of media) onto KC cells, as previously described.^18^

### ELISA

Serum BTV VP7 antibodies were detected with an ID Screen BT competition ELISA (Innovative Diagnostics) according to the manufacturer's instructions. IgM and IgG subclass‐specific BTV VP7 antibodies were detected, as previously described.^11^

### Serum neutralisation test

Neutralising (BTV viral protein 2 [VP2]) antibody titres were determined by serum neutralisation test, as previously described,^18^ using reference strain BTV‐3 SA [BHK5/V1] (The Pirbright Institute). Virus‐induced cytopathic effects in Vero cell monolayers (European Collection of Authenticated Cell Cultures) were recorded after 7 days of incubation (37°C, 5% CO_2_).

### Haematology

Complete blood counts were analysed using a ProCyte Dx Haematology Analyser (IDEXX Laboratories) according to the manufacturer's instructions.

## RESULTS

### Infection of sheep with an emerging UK BTV‐3 isolate using *C. sonorensis* midges

Similar numbers of *C. sonorensis* fed on each sheep; on average, 23 individuals (range 16‒29) (Table 1). Between 83% and 100% (average 92%) of those engorged or partially engorged midges fed on each sheep were considered to have a transmissible infection (*C*_q_ < 25; Table 1). The average *C*_q_ value for all engorged and partially engorged midges was 19.25 and all were BTV RNA positive at the time of feeding (Table 1).

**TABLE 1:** Numbers of engorged and partially engorged bluetongue virus serotype 3 inoculated *Culicoides sonorensis* that fed on each sheep to establish infection and the proportion with a transmissible infection

	Number of engorged midges	Number of bluetongue virus RNA‐positive engorged midges (%)	Mean *C*_q_ value of engorged midges (range)	Number of engorged midges with *C*_q_ < 25 (%)
Sheep 1	24	24 (100%)	20.55 (17.74‒33.06)	20 (83%)
Sheep 2	29	29 (100%)	19.04 (17.10‒33.02)	27 (93%)
Sheep 3	16	16 (100%)	19.95 (16.92‒33.55)	14 (88%)
Sheep 5	27	27 (100%)	18.57 (17.10‒31.82)	26 (96%)
Sheep 6	21	21 (100%)	18.40 (17.76‒19.38)	21 (100%)

### Clinical observations of BTV‐3 UKG2023 infection in British sheep

**FIGURE 2:**
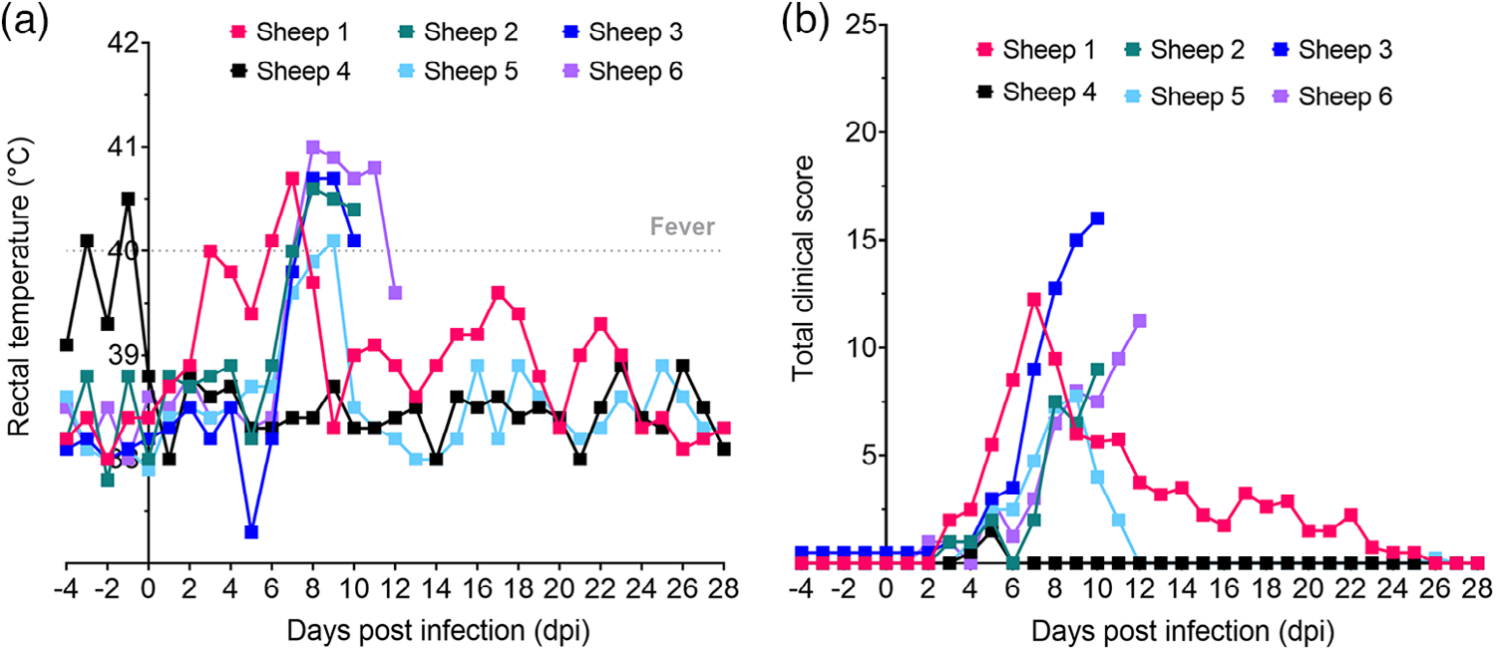
Clinical scoring of bluetongue disease in five British sheep during bluetongue virus serotype 3 strain UKG2023 infection. (a) Rectal temperatures (°C) and (b) total daily clinical scores. Sheep four was included as a negative (uninfected) control, and while mild fever was detected at pre‐infection time points, fever was not detected at any point from 0 to 28 dpi

**FIGURE 3:**
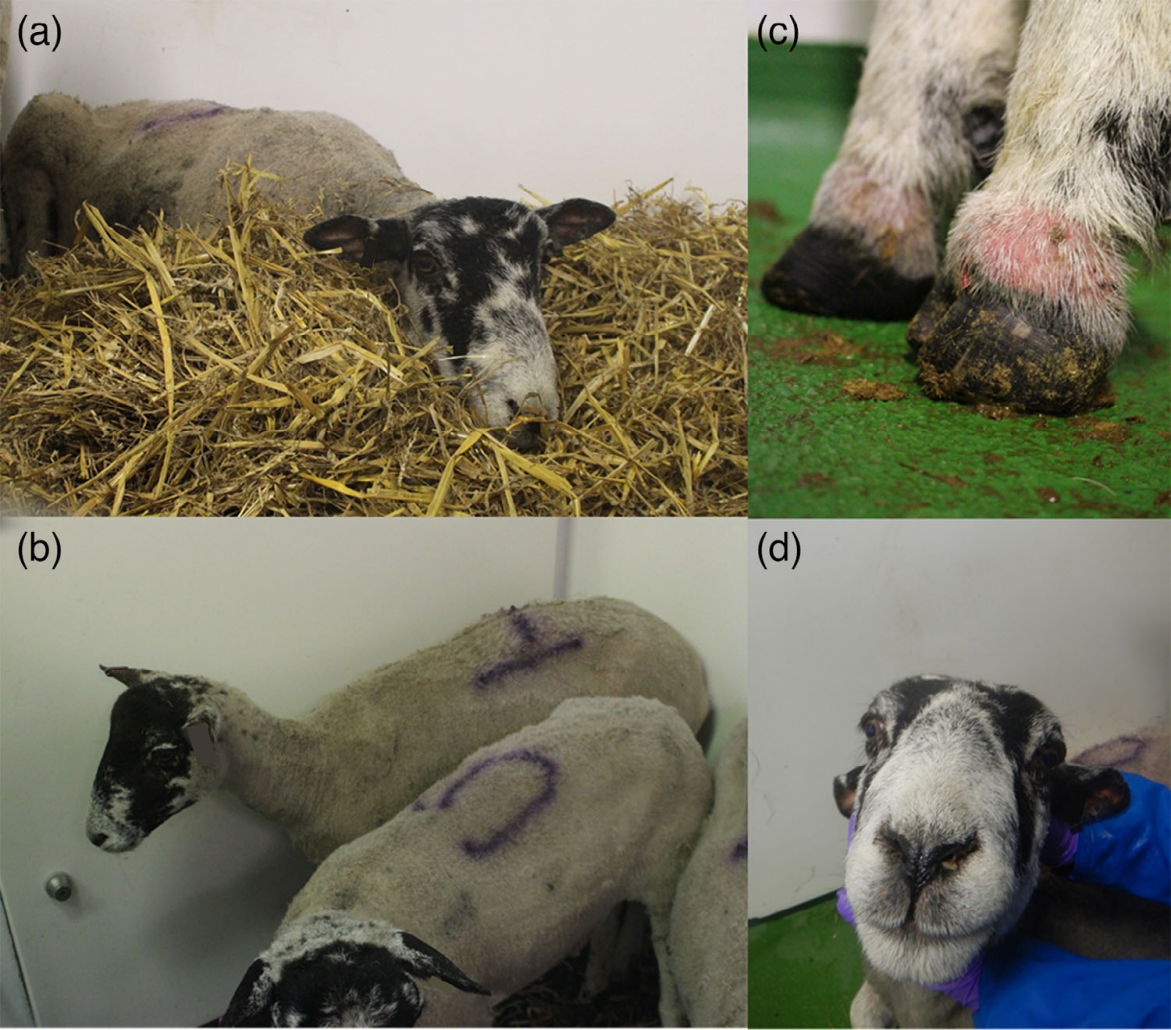
Clinical signs observed in British sheep during bluetongue virus serotype 3 strain UKG2023 infection. (a) Depression and reluctance to move, (b) shivering and depression with an arched back, (c) reddening and swelling of the coronary band (coronitis) and immediate proximal haired skin and (d) nasal discharge and mild facial oedema

All five BTV‐3‐infected sheep exhibited typical clinical signs of BT disease, including elevated rectal temperature, nasal discharge and reddening of the conjunctiva, oral mucosa and coronary band (Figures 2 and 3; Supporting Information S2). The clinical signs and severity of disease were variable between individuals (Figures 2 and 3), ranging from mild to moderate. Four of the five infected sheep developed marked fever, and temperature remained elevated in three sheep from 8 to 10 dpi (sheep two and three) or 11 dpi (sheep six) (Figure 2a). Sheep five progressed only to mild fever at 9 dpi (Figure 2a). The uninfected control (sheep four) had mild fever at ‒3 and ‒1 dpi (pre‐infection), indicating a likely pre‐existing bacterial/viral infection.

Sheep six progressively declined, becoming depressed and reluctant to move (Figure 3a), and was euthanased at 12 dpi, having reached a humane endpoint. For sheep one, fever was accompanied by behavioural signs of shivering, depression and an arched back (Figure 3b). Fever and pain were alleviated through NSAID treatment; however, this sheep later exhibited lameness associated with swollen and reddened coronary bands (Figure 3c). Sheep two and three developed haemorrhagic diarrhoea (a humane endpoint) at 10 dpi and were euthanased. Facial oedema was observed, most obviously in sheep three and six; however, it was mild (Figure 3d). All infected sheep exhibited reddened oral and ocular mucosa of mild severity. Mild nasal discharge was observed in sheep three (Figure 3d). Sheep four, the uninfected control, was clinically healthy throughout except for a mild non‐descript nasal discharge between 4 and 5 dpi and mild reddened mucosa at 5 dpi (Supporting Information S2).

### Viraemia dynamics varied during BTV‐3 UKG2023 infection in British sheep

**FIGURE 4:**
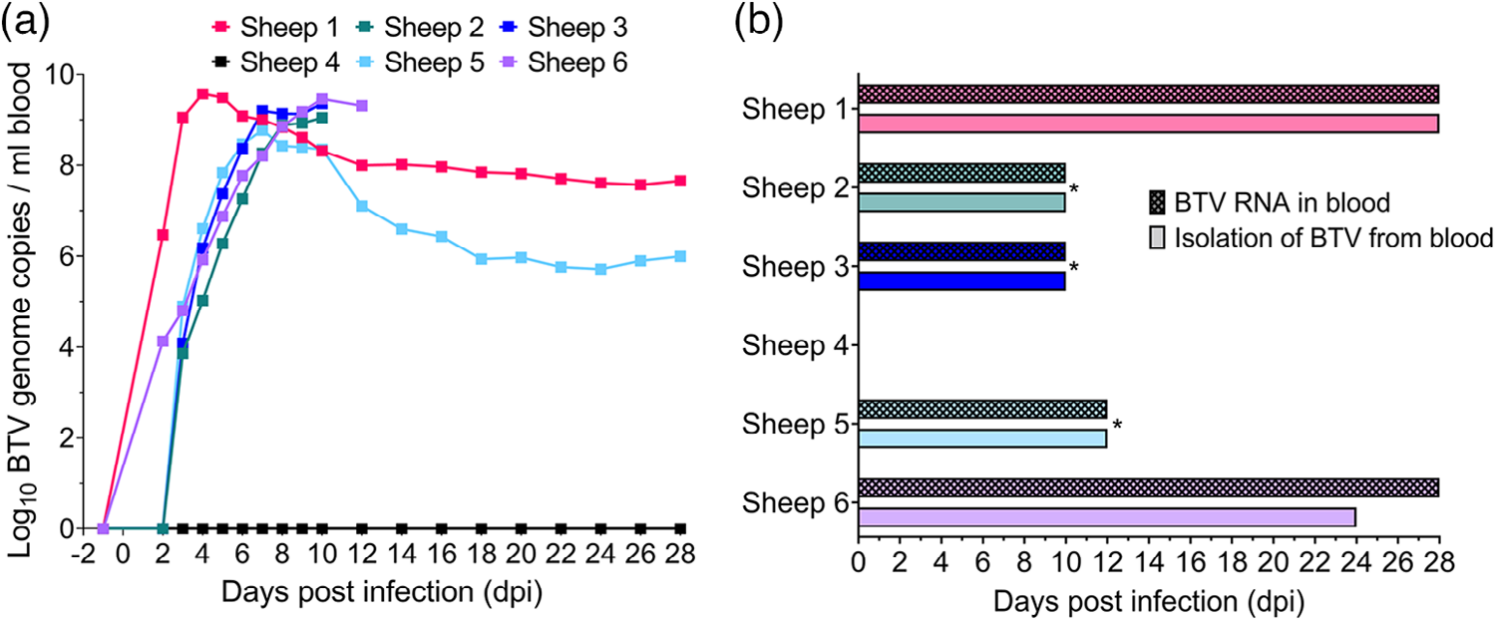
Dynamics of bluetongue virus serotype 3 (BTV‐3) strain UKG2023 infection in British sheep. (a) Log_10_ BTV genome copies per millilitre of blood in five British sheep during BTV‐3 UKG2023 infection. BTV RNA was not detected in the co‐housed negative (uninfected) control sheep (black), confirming that contact transmission had not occurred. (b) Comparison of detectable BTV RNA in the blood with the ability to isolate infectious BTV from the blood of British sheep during infection with BTV‐3 UKG2023. Asterisks denote euthanasia of sheep for reaching humane endpoints of the study; virus isolation was not attempted beyond this time point

BTV‐3 infection of all five British sheep was successful, with all developing viraemia (Figure 4a). BTV RNA was detectable in all sheep from 2 or 3 dpi; however, viraemia dynamics varied between individuals during infection (Figure 4a). Sheep one developed a rapid onset of viraemia, with BTV RNA levels reaching 6.47 log_10_ BTV genome copies per millilitre by 2 dpi, which was similar to the levels in other sheep at 4‒5 dpi. The timing of peak viral genome load varied between individuals, with sheep one peaking at 4 dpi and others peaking much later at 7 dpi (sheep five) and 10 dpi (sheep two, three, six) (Figure 4a). Despite this, all sheep had similar peak BTV RNA levels in the blood, averaging 9.24 (range 8.77‒9.57) log_10_ BTV genome copies per millilitre. However, BTV RNA levels were much lower at 28 dpi in sheep five compared to sheep one (5.99 and 7.66 log_10_ BTV genome copies per millilitre, respectively). Sheep four (uninfected control) remained BTV RNA negative throughout the study, indicating that no contact transmission had occurred (Figure 4a).

### UK BTV‐3 strain isolated from sheep blood at 28 days postinfection

BTV‐3 was isolated from the blood of both sheep (one and three) with detectable BTV RNA levels at 2 dpi, and from four of the five infected sheep (one, two, three, five) at 3 dpi, despite most having low BTV RNA levels in the blood (Supporting Information S3). BTV‐3 was isolated from the blood of all five infected sheep from 4 to 10 dpi (Supporting Information S3) and up until the humane endpoint of sheep six at 12 dpi (Figure 4b). Of the two sheep that survived to 28 dpi, BTV was isolated from the blood of sheep one at every time point tested (2‒28 dpi); however, BTV was only isolated from 3 to 24 dpi in sheep five (Figure 4b). This coincided with the lower BTV RNA levels detectable in the blood of sheep five at the end of the study compared to sheep one, suggesting a potential correlation with blood viral load (Figure 4a). The North American *Culicoides* species, *C. sonorensis*, was found to have a very low susceptibility to this UK BTV‐3 strain (Supporting Information S4); however, this is unlikely to reflect infection rates of UK/Palearctic *Culicoides* species.

### BTV‐3 infection causes panleukopaenia in British sheep around peak viraemia

Total leukocyte numbers declined in all infected sheep around peak viraemia, typically between 7 and 12 dpi, but earlier (4‒9 dpi) for sheep one (Supporting Information S5). This was driven by a decline in lymphocytes, eosinophils, basophils and sometimes neutrophils (Supporting Information S5). Sheep five had a substantially higher preinfection neutrophil count than other sheep, which rapidly declined from 4 dpi and was thereafter maintained at lower levels to 28 dpi (Supporting Information S5). This sheep also had a greater eosinophil and basophil recovery to 28 dpi compared to sheep one. Red blood cell counts were maintained throughout infection in all sheep (data not shown).

### Antibody dynamics were not uniform in BTV‐3‐infected sheep

**FIGURE 5:**
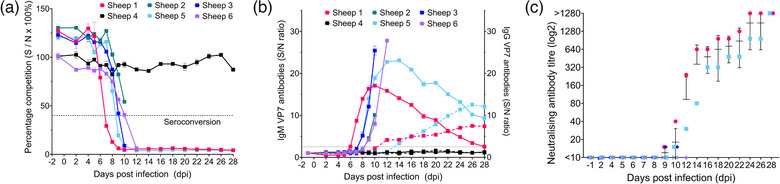
Bluetongue virus (BTV) antibody dynamics in British sheep during BTV serotype 3 strain UKG2023 infection. (a) Detection of BTV viral protein 7 (VP7) antibodies, with seroconversion defined by percentage competition (sample optical density [OD]/negative OD [S/N] × 100%) below 40. (b) Immunoglobulin M (IgM) (solid) and IgG (hashed) BTV VP7 antibody dynamics expressed as a sample OD/negative (preinfection) OD (S/N) ratio. Values are means of duplicate technical replicates (error bars, ±SD). (c) Log_2_ (±SD) neutralising anti‐BTV viral protein 2 (VP2) antibody titres in five BTV‐3‐infected sheep (sheep one, pink; sheep two, green; sheep three, dark blue; sheep five, light blue; sheep six, purple). Sheep four (negative control; black) remained uninfected throughout the study

Four of the five infected sheep had detectable BTV VP7 antibodies by diagnostic ELISA (Figure 5a). Sheep two did not seroconvert before euthanasia (10 dpi). Time to seroconversion was variable, with sheep five seroconverting at 9 dpi, sheep three at 10 dpi and sheep six at 12 dpi (Figure 5a). In line with its earlier onset of viraemia, sheep one seroconverted much earlier at 7 dpi (Figure 5a). These antibodies were of the IgM subclass (Figure 5b), peaking at 10 dpi (sheep one) and 14 dpi (sheep five) and declining to 28 dpi. IgG VP7 antibodies were only detectable in sheep one and five, from 12 and 14 dpi, respectively, and peaked and plateaued in both from 26 dpi (Figure 5b).

Protective neutralising antibodies were only detected in three of the five infected sheep (Figure 5c), from 9 dpi (sheep one and three) and 10 dpi (sheep five), respectively. Sheep one reached antibody titres greater than 1:1280 earlier (24 dpi) than sheep five (28 dpi) (Figure 5c). While sheep one and five had neutralising antibody titres of 1:240 and 1:30, respectively, at 12 dpi, neutralising antibodies were not detected in sheep six at 12 dpi (Figure 5c).

### Gross pathology of BTV‐3 UKG2023 infection in British sheep

**FIGURE 6:**
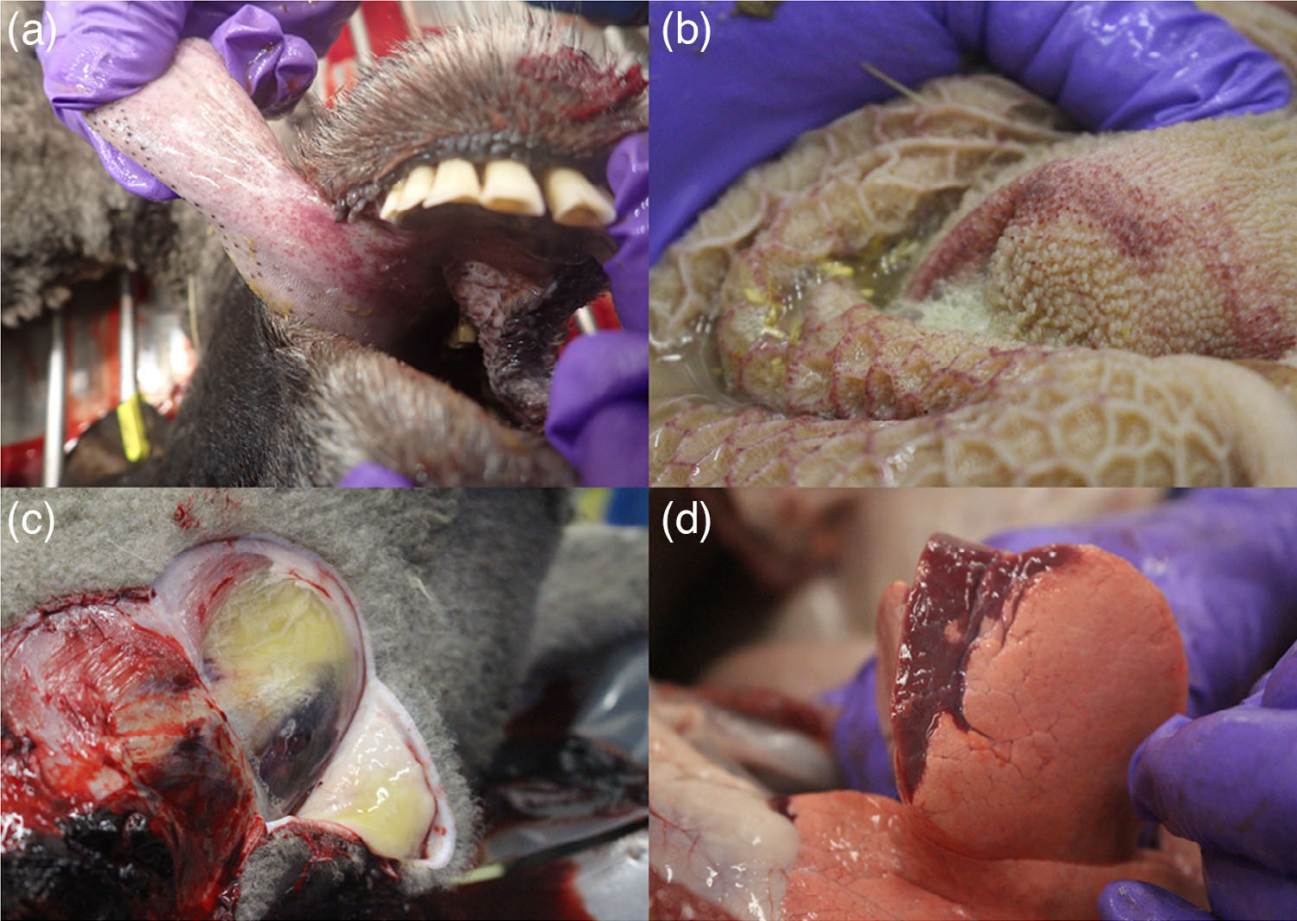
Gross pathology postmortem in British sheep experimentally infected with bluetongue virus serotype 3 strain UKG2023. Petechial haemorrhages on the (a) underside of the tongue and (b) reticulum, (c) subcutaneous oedema and (d) congestion of the lung

Postmortem examination of sheep two and three at 10 dpi and sheep six at 12 dpi revealed extensive sublingual petechial haemorrhage as a consistent abnormality (Figure 6a). To a lesser extent, petechial haemorrhage was observed on the surface of the larynx and cut surface of the palatine tonsil in these sheep. Sheep six had mild petechiation on the nasal mucosae proximal to the nares, proximal oesophagus and trachea and conjunctiva. Petechia were inconsistently observed on the cut surface of some lymph nodes (submandibular, prescapular, parotid and retropharyngeal) in all three sheep. The gastrointestinal tract distal to the oesophagus had mild petechial haemorrhage on the luminal surface of the ascending (sheep two) and descending (sheep six) colon. Sheep six also had petechiation on the luminal surfaces of the abomaso‐duodenal (combined with ecchymosis) and reticulo‐omasal junctions, rumen and reticulum (Figure 6b). Sheep six had lines of petechiae along the distal descending colon and rectum. Only sheep six demonstrated the characteristic subcutaneous oedema (Figure 6c) of clinical BT, ventral to the chin and lateral to the neck and flank. Sheep two had excess fluid (>30 mL) in the abdominal cavity (ascites). Lung changes were observed in all three sheep, with mild reddening and small areas of petechiae on lung lobe surfaces in sheep two and three, respectively. Sheep six demonstrated marked consolidation of the right cranial, middle and accessory lobes and borders of the left cranial lobe (Figure 6d). Sheep six had an enlarged spleen with ‘wet’ cut surfaces and rounded edges, and its kidneys were reddened at the corticomedullary junction, with diffuse areas of round discolouration on the surfaces.

By 28 dpi, gross pathology of BTV‐3 infection in sheep one and five was comparatively reduced; however, both had developed lip lesions and minor sublingual and/or gingival petechiae. Sheep five had several red nodules on the spleen surface and some marked subendocardial discolouration of the left ventricle. All sheep (except sheep three) had moderately increased pericardial fluid (6‒14 mL). BTV RNA was detectable in nearly all target organs/tissues of infected sheep postmortem (Supporting Information S6).

## DISCUSSION

The detection of BTV‐3 in UK ruminants in late 2023 elicited an urgent need to determine the clinical impact and infection kinetics in UK sheep to assess the potential risk of re‐emergence or another incursion. The BTV‐3 strain responsible for the 2023 UK incursion (UKG2023) was isolated from the blood of an infected cow and, due to a low initial infectious viral titre, was minimally passaged to achieve a sufficient titre to facilitate infection of sheep through *C. sonorensis* midges. Despite four passages through the *Culicoides‐*derived KC cell line, the virus remained representative of the original BTV‐3 isolate through confirmed sequence identity across all 10 BTV genome segments.

All five sheep were infected with the BTV‐3 UKG2023 isolate, eliciting mild to moderate clinical BT disease, with some parallels to signs reported in the Netherlands outbreak.^8^ Clinical signs of disease varied in type and severity between individuals, with three infected sheep reaching humane endpoints due to the onset of haemorrhagic diarrhoea or marked and ongoing behavioural changes (depression and isolation). Of the two remaining infected sheep, one avoided early euthanasia following response to NSAID treatment. It is highly likely that the three euthanased sheep (reaching humane endpoints between 10 and 12 dpi) would otherwise have progressed to develop more severe clinical disease and, under field conditions, may have naturally succumbed to infection, particularly if undetected and untreated. In contrast, one sheep exhibited only mild clinical signs of disease. Pyrexia was a factor common to all sheep and may have continued in four sheep without intervention. Classical BT clinical signs and gross pathology were observed, including facial and subcutaneous oedema, reddened mucosal membranes, prominent and consistent petechial haemorrhaging characteristic of vascular endothelial cell damage, and widespread infection of target organs/tissues. The one consistent clinical sign in all five infected sheep was sublingual petechial haemorrhage. However, these observations were milder than previously observed in other sheep breeds and with other BTV strains.^1^, ^11^, ^15^, ^21^

During the UK BTV‐3 incursion, clinical signs were not reported in any infected sheep or cattle at the time of sampling,^4^ directly contrasting the severe clinical disease reported in ruminants, particularly sheep, during the recent Netherlands BTV‐3 outbreak.^8^ The sequence identity of the BTV‐3 NET2023 and UKG2023 isolates confirmed that differences in pathogenicity are likely not due to viral genetic differences. The lack of reported BT in the UK may result from missed clinical signs in the field, breed‐specific differences in clinical manifestation between Dutch and British sheep, or simply reflect the low number of infected sheep detected. The clinical signs observed in this study could easily have gone undetected unless animals were under close and continuous observation. Behavioural isolation and reluctance to move may provide an indicator of fever or other signs of BTV infection. North Country mule sheep (blue‐faced Leicester × Swaledale) were used in this study as they are a common breed found across the UK. Other sheep breeds, particularly Dorset/Texel crosses, may develop more severe clinical signs of BT, which may explain why some clinical signs reported in Dutch sheep were not observed here, including hypersalivation and erosions of the oral/nasal mucosa.^8^

The infection dynamics and immune response to BTV‐3 were highly variable between sheep; however, they generally followed those elicited by European BTV‐4 and BTV‐8 strains.^11^, ^21^ Peak BTV RNA levels were comparable to sheep infected with BTV‐4 MOR2009/07, but were higher than in sheep infected with several BTV‐8 strains. The timing and magnitude of detectable BTV RNA and clinical disease were not correlated with the number of bites received from infected *Culicoides* in any of the sheep. BTV VP7 antibodies were consistently detected from 2 to 3 days post‐peak viraemia, as previously observed with BTV‐4 MOR2009/07 and BTV‐8 UKG2007 strains, with an initial IgM subclass followed by later class switching to IgG at around 12‒14 dpi.^11^, ^21^ Neutralising antibodies against BTV VP2, which are the only known correlate of protection for ruminants against homologous BTV infection, were detected in three of the infected sheep from 9 or 10 dpi, including both that survived to 28 dpi. Neutralising antibody titres in infected sheep initially followed similar levels to those reported for BTV‐8‐infected sheep^21^; however, they increased to greater antibody titres by 21 dpi. All BTV‐3‐infected sheep demonstrated the characteristic panleukopaenia of BTV infection at peak viraemia.^25^ Interestingly, sheep five, the only clinically mild sheep, had a comparatively greater preinfection neutrophil count than the other sheep, suggesting a potential role for neutrophils in individual variation in disease severity.

Infectious BTV‐3 was isolated from the blood of sheep for 28 dpi, longer than previously reported in sheep infected with other BTV strains (10‒17 dpi for several BTV‐8 and BTV‐17 strains).^19^, ^21^, ^26^ Isolation of infectious BTV‐3 appeared to correlate with relative BTV RNA levels in the blood, with sheep one demonstrating a prolonged infectious viraemia and higher BTV RNA levels at later time points than sheep five. It would be epidemiologically important to understand whether BTV‐3 remains infectious in the blood of sheep beyond 28 dpi and whether this is observed for other domestic/wild ruminants, particularly cattle. The preliminary data suggest that this BTV‐3 strain may have a more prolonged window for *Culicoides* transmission than other studied European BTV strains, but this requires further investigation. Work is also needed to assess the susceptibility of native UK midges to BTV‐3 UKG2023 to define transmission parameters.

## CONCLUSIONS

Over 15 years since the last BTV incursion, an emerging European BTV‐3 isolate was detected in the UK and has been shown to cause mild to moderate clinical disease in British sheep, confirming that a future BTV‐3 incursion or outbreak could have potentially devastating consequences for our naïve sheep population. It is now known that BTV‐3 can remain infectious in the blood of sheep for at least 28 dpi, but the period during which an animal remains infectious to *Culicoides* vectors has yet to be determined. The clinical impact for other domestic and wild ruminants, particularly cattle, has also yet to be investigated. BTV‐3 vaccines are now licensed for emergency use in Europe, however not currently in the UK, therefore the emphasis for BTV control in UK livestock populations remains with close monitoring of animals and rapid reporting of clinical BT suspicions.

## AUTHOR CONTRIBUTIONS

Data curation, formal analysis, investigation, methodology, project administration, resources, supervision, validation, visualisation, writing—original draft and reviewing/editing: Kerry Newbrook. Data curation, formal analysis, investigation, validation and writing—reviewing/editing: Emmanuel Obishakin. Investigation and writing—reviewing/editing: Laura A. Jones. Investigation, resources, supervision and writing—reviewing/editing: Ryan Waters. Formal analysis, investigation, supervision, validation and writing—reviewing/editing: Martin Ashby. Conceptualisation, funding acquisition, methodology, project administration, resources, supervision and writing—reviewing/editing: Carrie Batten. Conceptualisation, funding acquisition, investigation, methodology, project administration, resources, supervision, visualisation, writing—original draft and reviewing/editing: Christopher Sanders.

## CONFLICT OF INTEREST STATEMENT

The authors declare no conflicts of interest.

## ETHICS STATEMENT

This study was carried out in accordance with The Animals (Scientific Procedures) Act 1986 (ASPA) and the European Directive 2010/63/EU, which is transposed into UK law by ASPA. The study was conducted under Project Licence PP5796660, approved by the UK Home Office, and locally reviewed and approved by the Animal Welfare and Ethical Review Body at The Pirbright Institute.

## Supporting information



Supporting Information

## Data Availability

The data that support the findings of this study are available in the Supporting Information of this article.
