# Supplementary material for: Clinical disease in British sheep infected with an emerging strain of bluetongue virus serotype 3
Source: Vet Rec. 2024 Dec 16;196(4):e4910. doi: 10.1002/vetr.4910 (PMC11827624; doi:10.1002/vetr.4910)
Supplement: Supplementary file 1 — Supporting Information [file VETR-196-e4910-s001.pdf]

Supporting information

Supplementary Data 1

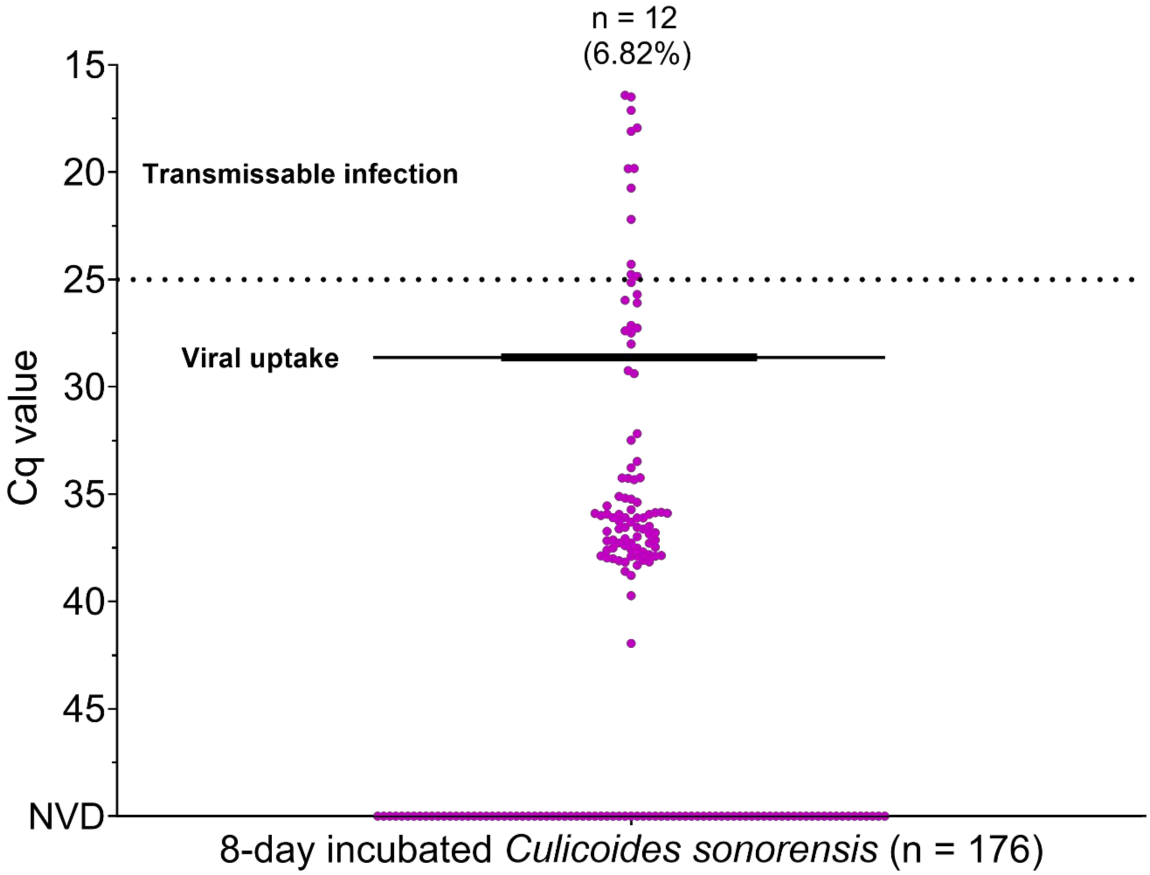

Figure S1. Infection rate of *Culicoides sonorensis* for bluetongue virus serotype 3 strain UKG2023 by oral membrane feeding. *Culicoides sonorensis* were orally blood-fed bluetongue virus (BTV) serotype 3 strain UKG2023 through a membrane at a concentration of $1x10^7$ TCID$_{50}$ per millilitre to determine their infection rate. BTV RNA levels (denoted by C_q values) were quantified in engorged *C sonorensis* following incubation for eight days (D8; n=176; purple dots) to determine those with a transmissible infection (C_q < 25; 6.82%, n=12). Baseline viral uptake (median and range, black lines) was determined by quantifying BTV RNA levels in a subset (n=16) of these individuals immediately following feeding (D0).

Supplementary Data 2

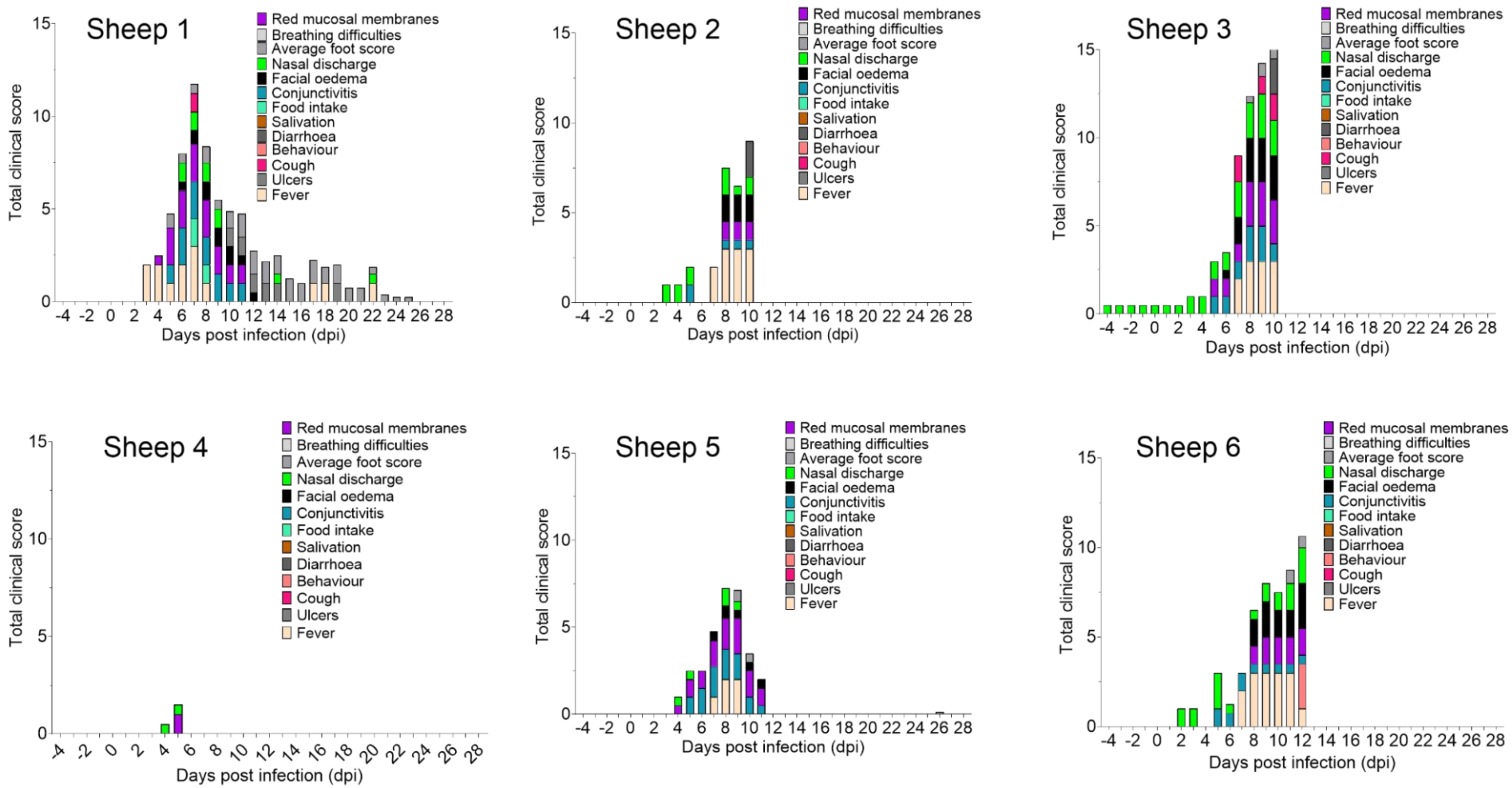

Figure S2. Total clinical scores demonstrating the range and dynamics of clinical signs observed in British sheep during bluetongue virus serotype 3 UKG2023 infection. British sheep demonstrated several typical bluetongue virus-associated clinical signs during bluetongue virus serotype 3 UKG2023 infection, with individual variation in signs and severity ranging from mild to severe clinical disease. The negative control (sheep four) remained uninfected and clinically healthy during the study except for a mild nasal discharge at 4 and 5 dpi and mild reddening of the mucosa at 5 dpi.

Supplementary Data 3

Table S3. Isolation of bluetongue virus serotype 3 from EDTA blood of British sheep during infection. To determine the duration that bluetongue virus serotype 3 (BTV-3) strain UKG2023 remained infectious in the blood of the five infected sheep, virus isolation was attempted from EDTA blood of each sheep at each day post infection (where BTV RNA was detectable) through passage onto KC cells. BTV RNA levels were compared in the original EDTA blood sample, the inoculum (1:10 dilution of washed and sonicated blood) and the harvested material following incubation to allow viral infection/replication. BTV was isolated if the change in C_q value from inoculum to harvest was less than three. If no virus was detected (NVD) in the harvest material, virus isolation was unsuccessful and repeated once more from the original material (*). If the C_q value dropped from inoculum to harvest but by less than $C_q < 3$, the harvest material was re-passaged onto KC cells (†) and reassessed. Successful (green), unsuccessful (red), doubtful (amber).

Days post infection	Sheep 1			Sheep 2			Sheep 3			Sheep 5			Sheep 6		
	EDTA	Inoculum	Harvest	EDTA	Inoculum	Harvest	EDTA	Inoculum	Harvest	EDTA	Inoculum	Harvest	EDTA	Inoculum	Harvest
**2**	28.81	34.80	19.83				39.37	NVD	21.31*						
**3**	19.99	23.92	15.71	37.71	NVD	20.01*	36.95	35.40	30.14*	34.17	35.49	22.57*	34.50	NVD	39.13*†
**4**	18.22	23.02	16.29	33.73	38.17	24.94	29.81	32.58	23.67	28.34	31.97	18.55	30.67	35.75	20.16
**5**	18.50	22.90	15.85	29.47	32.45	18.85	25.72	28.14	16.91	24.13	27.50	17.01	27.42	32.46	19.41
**6**	19.87	24.21	16.15	26.11	28.97	15.76	22.32	25.19	16.50	22.00	25.59	15.54	24.35	29.41	16.86
**7**	20.17	24.90	15.77	22.67	25.97	15.38	19.49	23.54	16.45	20.95	25.49	15.90	22.87	27.27	16.10
**8**	20.71	25.81	16.74	20.57	24.14	15.56	19.70	23.04	16.26	22.12	25.92	15.52	20.66	25.49	16.02
**9**	21.49	25.95	11.36	20.40	23.37	8.62	19.72	23.40	10.56	22.22	26.13	10.32	19.56	24.40	9.66
**10**	22.50	27.75	11.86	20.00	23.05	8.97	18.94	22.78	11.04	22.38	26.84	12.11	18.60	22.94	9.76
**12**	23.59	28.49	20.40							26.64	31.43	25.42	19.11	23.66	9.97
**14**	23.53	28.13	15.70							28.38	32.10	26.73			
**16**	23.70	27.60	19.49							28.93	30.87	23.23*			
**18**	24.10	28.17	19.43							30.63	31.47	23.72*			
**20**	24.20	28.46	22.85							30.51	31.17	27.31*			
**22**	24.58	24.25	14.14†							31.22	31.52	27.11			
**24**	24.91	28.03	14.19							31.39	31.20	18.73*			
**26**	25.04	28.89	18.89							30.76	31.31	NVD*			
**28**	24.75	28.19	19.34							30.43	NVD	NVD†			

Supplementary data 4

Low infection rate of *Culicoides sonorensis* blood fed on peak viraemic sheep during bluetongue virus serotype 3 UKG2023 infection. The highly characterised *Culicoides sonorensis* colony was used as a model vector to determine the rate of infection in naïve *Culicoides* midges fed on British BTV-3 infected sheep at their peak viremia, performed as previous described.[15] Whilst not native to the UK, and endemic only to North America, this *Culicoides* species are highly established and widely used for *in vivo* BTV infection and transmission studies in large ruminants.[11,15,19] Following extrinsic incubation (25°C, eight days) of the blood-fed midges, surviving *Culicoides* were dissected into head and body (50 individuals per sheep) or left whole prior to homogenisation, RNA extraction and quantification of BTV RNA to assess vector infection rate. Sixteen blood fed *Culicoides* per sheep were processed immediately after feeding to quantify baseline viral uptake.

When assessing baseline viral uptake, BTV RNA was detectable in all but two of the 56 engorged *C. sonorensis* midges fed on the peak viraemic sheep infected with BTV-3 UKG2023, with average C_q values of 31.34 (sheep one), 32.33 (sheep two), 31.08 (sheep three), 33.04 (sheep five) and 35.13 (sheep six) respectively (range 30.11-37.43). However, in nearly all *C sonorensis* tested, we detected no dissemination of infection following extrinsic incubation (D8). BTV RNA was not detectable in any of the whole bodies and only at very low levels (C_q 41.21) in one dissected *C sonorensis* body of an individual fed on sheep two. BTV RNA was detectable in the heads of each one individual fed on sheep one, two and six, however again at levels which are considered unlikely to be transmissible (C_q 32.71, 34.15, 38.26 respectively).

Whilst BTV-3 was consistently taken up in the blood-meal of naïve *C sonorensis* when feeding on viraemic sheep, our study found that the infection rate of this non-native *Culicoides* species was very low, similar to that of the BTV-8 NET2006/04 strain.[14]

Supplementary data 5

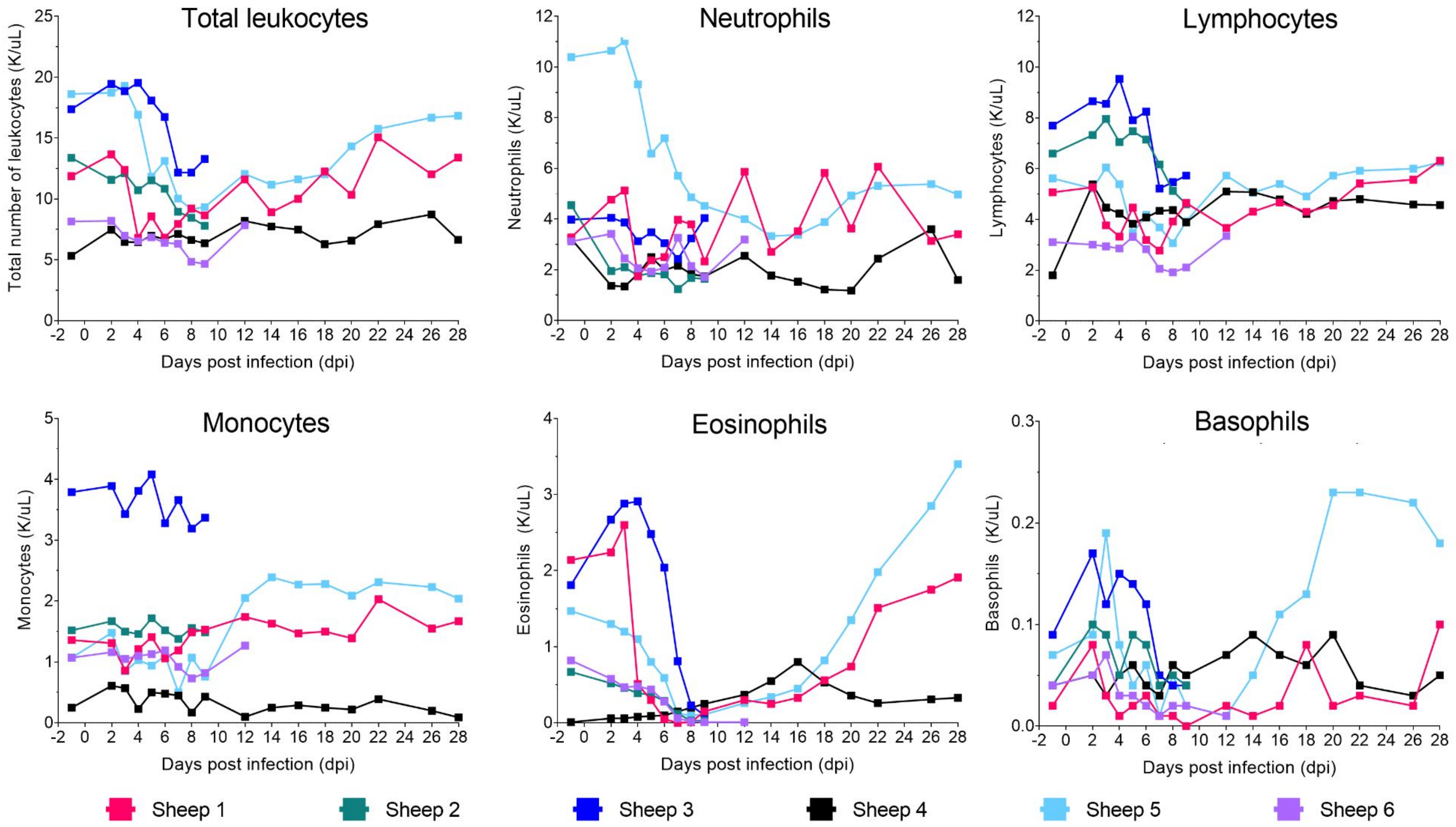

Figure S5. Dynamics of complete immune cell counts during bluetongue virus serotype 3 UKG2023 infection in five British sheep. Complete counts of **(A)** total leukocytes, **(B)** neutrophils, **(C)** lymphocytes, **(D)** monocytes, **(E)** eosinophils and **(F)** basophils are denoted as x1000 cells per microlitre of EDTA blood. Sheep four was included as a negative control animal and remained uninfected throughout the study.

Supplementary data 6

BTV RNA is detectable in nearly all target organs and tissues of BTV-3 UKG2023 infected sheep. BTV RNA was detectable in all tissues and organs surveyed from the BTV-3 sheep except for the mesenteric lymph node (Table S6). Viral loads were greatest in the tissues of sheep euthanized earlier during infection (10 dpi for sheep two and three, 12 dpi for sheep six), however also appeared to corelate to viral load in the blood as BTV RNA levels were consistently higher in sheep one than sheep five (Table S6: Fig 4A). Although detectable in all other infected sheep, BTV RNA was not detected in the liver, spleen or superficial inguinal lymph node of sheep five. BTV RNA was only detectable in the abomasum-duodenum junction of one of the three sheep tested, notably sheep six which was the only one of the three euthanized earlier during infection. BTV RNA was detectable in coronary band tissue, however only in the two sheep surviving to 28 dpi (sheep one and five). Interestingly, all lip lesion tissues were found to be BTV RNA positive, as well as abnormal sub-oedematous fluid from sheep six and abnormal pericardial fluid from three of four sheep for which it was tested (Table S6).

Table S6. Detectable RNA levels (C_q) of bluetongue virus serotype 3 (UKG2023) in tissues and organs of infected sheep postmortem.

Tissue / cavity fluid	Sheep 1	Sheep 2	Sheep 3	Sheep 4	Sheep 5	Sheep 6
Ear	30.69	27.59	25.03	NT[*]	33.48	27.37
Lip (lesion)	37.20	NT[*]	NT[*]	NT[*]	38.11	NT[*]
Tongue	35.37	24.64	NT[*]	NVD[*]	36.06	NT[*]
Tonsil	36.65	42.35	27.79	NT[*]	44.19	26.08
Heart	33.41	23.62	23.55	NVD[*]	34.98	25.92
Lung	NVD[*]	NVD[*]	25.80[†]	NVD[*]	42.91[†]	27.69[†]
Kidney	36.33	25.41	26.52	NVD[*]	38.13	30.63
Liver	41.44	27.97	28.46	NVD[*]	NVD[*]	25.31
Spleen	34.73	27.81	23.60	NVD[*]	NVD[*]	24.70
Duodenum-abomasum junction	NVD[*]	NT[*]	NT[*]	NT[*]	NVD[*]	25.07
Coronary band	33.98	NT[*]	NT[*]	NT[*]	34.11	NT[*]
Red bone marrow	34.80	28.98	25.78	NT[*]	42.40	32.73
Parotid gland	33.69	NT[*]	NT[*]	NVD[*]	33.90	NT[*]
Parotid lymph node	27.24	29.68	27.73	NVD[*]	32.81	30.34
Superficial inguinal lymph node	33.21	32.34	30.72	NVD[*]	NVD[*]	32.79
Mandibular lymph node	32.13	27.50	26.28	NT[*]	40.52	27.51
Mesenteric lymph node	NVD[*]	NVD[*]	NVD[*]	NT[*]	NVD[*]	NVD[*]
Prescapular lymph node	35.99	35.67	26.18	NT[*]	43.62	35.52
Tracheobronchial lymph node	35.31	NT[*]	NT[*]	NT[*]	37.84	29.68
Peri-bronchial lymph node	34.00	42.58	28.57	NT[*]	NVD[*]	NT[*]
Retropharyngeal lymph node	35.21	NT[*]	NT[*]	NT[*]	NVD[*]	31.87
Sub-oedematous fluid	NT[*]	NT[*]	NT[*]	NT[*]	NT[*]	27.97
Pericardial fluid	38.22	30.62	NT[*]	NVD[*]	NVD[*]	29.95
Abdominal cavity fluid	NVD[*]	31.82	NT[*]	NT[*]	NT[*]	NT[*]

[*]NT, not tested. NVD, no virus detected.

[†]C_q values achieved through 1:10 dilution of tissue homogenate in nuclease-free water (the equivalent undiluted sample was NVD).
